# Tracheobronchopathia osteoplastica: a clinical case report of a 63-year-old man

**DOI:** 10.3389/fmed.2024.1410724

**Published:** 2024-11-20

**Authors:** Jing Lai, Yingli Deng, Qingmin He

**Affiliations:** ^1^Second Ward, Department of Respiratory and Critical Care Medicine, Ankang Central Hospital, Ankang, China; ^2^Department of Gastroenterology, Ankang Central Hospital, Ankang, Shaanxi, China; ^3^Henan Key Laboratory of Helicobacter Pylori, Microbiota and Gastrointestinal Cancer, Marshall Medical Research Center, Fifth Affiliated Hospital of Zhengzhou University, Zhengzhou, China

**Keywords:** tracheobronchopathia osteoplastica, trachea, bronchus, case report, diagnosis

## Abstract

Tracheobronchopathia osteoplastica (TO) is a rare benign lesion of the airways, characterized by multiple nodular proliferations of bone and/or cartilaginous tissue in the submucosa of the trachea and bronchi. In this paper, we present a case of a 63-year-old male patient who was admitted to the hospital due to cough and blood in his sputum. The patient was diagnosed with TO through examination and pathological biopsy. The exact pathogenesis of this disease remains unclear, but it may be related to multiple factors such as chronic infection and inflammation. The clinical manifestations are diverse, with no symptoms or only mild cough in the early stage, and persistent cough, dyspnea, and other symptoms may appear as the disease progresses. Diagnosis mainly relies on pathological biopsy, while treatment is mainly symptomatic, including inhalation of glucocorticoids, airway interventional therapy, and stent placement. Overall, TO progresses slowly and has a good prognosis, but early diagnosis and elimination of predisposing factors are crucial for treatment.

## 1 Introduction

Tracheobronchopathia osteoplastica (TO) is an extremely rare benign lesion of the airways encountered in clinical practice (1). Its main pathological feature is the presence of multiple nodular proliferations of bone and/or cartilaginous tissue in the submucosa of the trachea and bronchi, which protrude into the lumen, forming specific lesion manifestations (1). Although most patients do not exhibit obvious symptoms or may only have a mild cough (2), bronchoscopic examination reveals multiple nodules on the anterior and lateral walls of the trachea and bronchi. These nodules, with diameters ranging from 1 to 5 millimeters, may be scattered or fused into patches, exhibiting morphological changes resembling pebbles or stalactites (3). In severe cases, these lesions may also involve the membranous portion and glottis region, providing important diagnostic clues. Given the rarity of the disease and the non-specificity of its symptoms, the diagnostic process is often challenging. However, early accurate diagnosis and treatment significantly impact the prognosis of patients. Therefore, deepening the understanding of TO is crucial. This article aims to enhance the medical community’s cognitive level of this disease by presenting a detailed case report of TO.

## 2 Case presentation

A 63-year-old male presented to our hospital with a two-year history of cough and intermittent blood-tinged sputum for 20 days. Two years prior, the patient developed paroxysmal irritating cough and expectoration without apparent provocation, unaccompanied by post-exertional wheezing or shortness of breath. Despite treatment at a local clinic, the cough and expectoration persisted for more than 3 months annually. Twenty days before admission, the patient noticed blood in his sputum after physical activity, without chest tightness or pain. He was subsequently hospitalized at Shiquan County Hospital, where relevant investigations did not support a diagnosis of tuberculosis. The patient was discharged after 1 week when the hemoptysis ceased, but the cough and expectoration continued intermittently. The patient presented to our outpatient clinic yesterday with recurrent blood-tinged sputum and was admitted for further evaluation of suspected pneumonia, chronic bronchitis, and the etiology of hemoptysis. Since the onset of symptoms, the patient denied fever, night sweats, fatigue, or weight changes. His past medical history was significant for a diagnosis of *Pseudomonas aeruginosa* pneumonia and hamartoma at Tangdu Hospital in 2017, treated with bronchoscopic surgery. He also had a two-year history of working in the coal mining industry. The patient had a history of smoking for 10 years, with a daily consumption of 2–3 cigarettes, and had quit smoking for 5 years. There were no similar medical records of tracheobronchial ossification among other family members. The patient was primarily engaged in farming, had lived locally for a long time, had no history of itinerant life, exposure to toxic gases or special chemicals, or adverse social history. Physical examination revealed hyperresonant lungs on percussion, coarse breath sounds on auscultation, and no abnormalities in the cardiovascular, abdominal, or neurological systems. The initial diagnoses were acute exacerbation of chronic bronchitis and hemoptysis of unknown etiology. Initial treatment included anti-infection therapy (piperacillin-tazobactam 4.5 g q8h), hemostasis (thrombin lyophilized powder 2,000 IU via nebulization bid), cough suppression and expectoration facilitation (Suhuang cough relief capsules 1.35 g tid, carbocisteine oral solution 25 mL tid).

**Figure 1:**
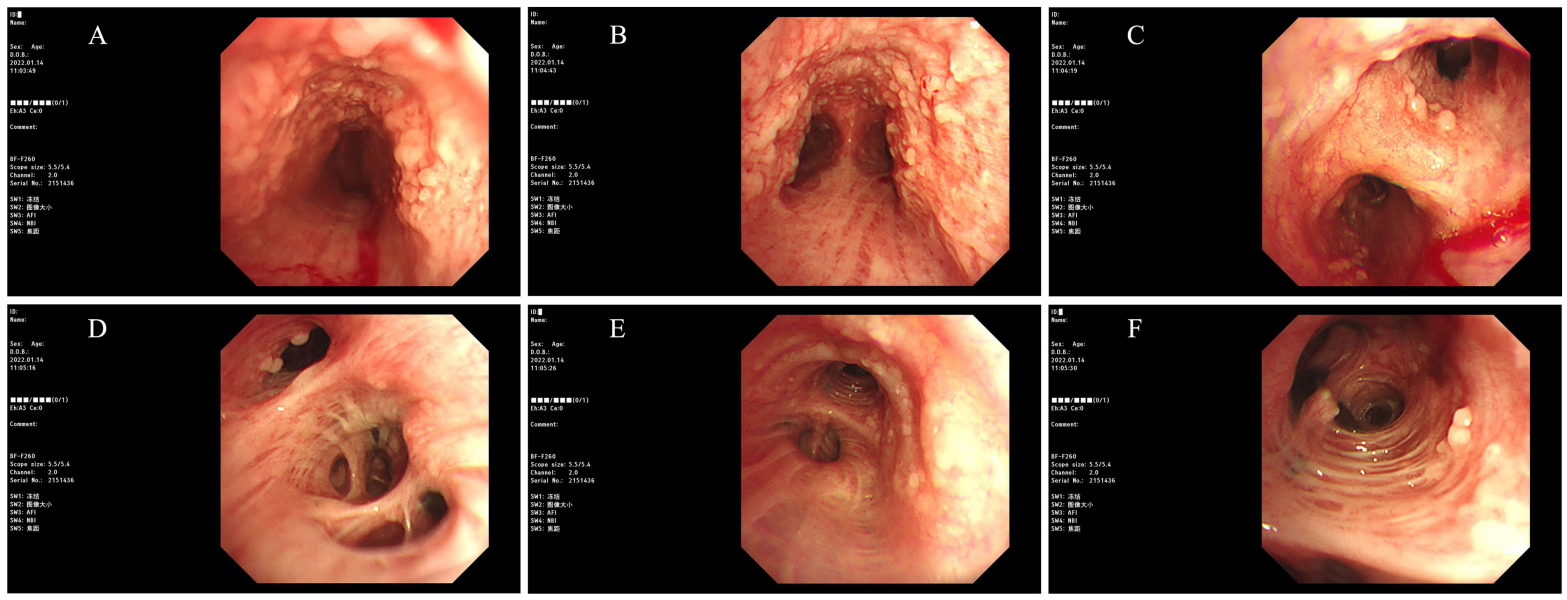
Images obtained under bronchoscopy (Bronchoscopy revealed nodular lesions of unknown etiology in the trachea and bilateral bronchi. (**(A)** Trachea, **(B)** Carina, **(C)** Right Main Bronchus, **(D)** Right Intermediate Bronchus, **(E)** Left Main Bronchus, **(F)** Left Upper Lobe Bronchus)).

**Figure 2:**
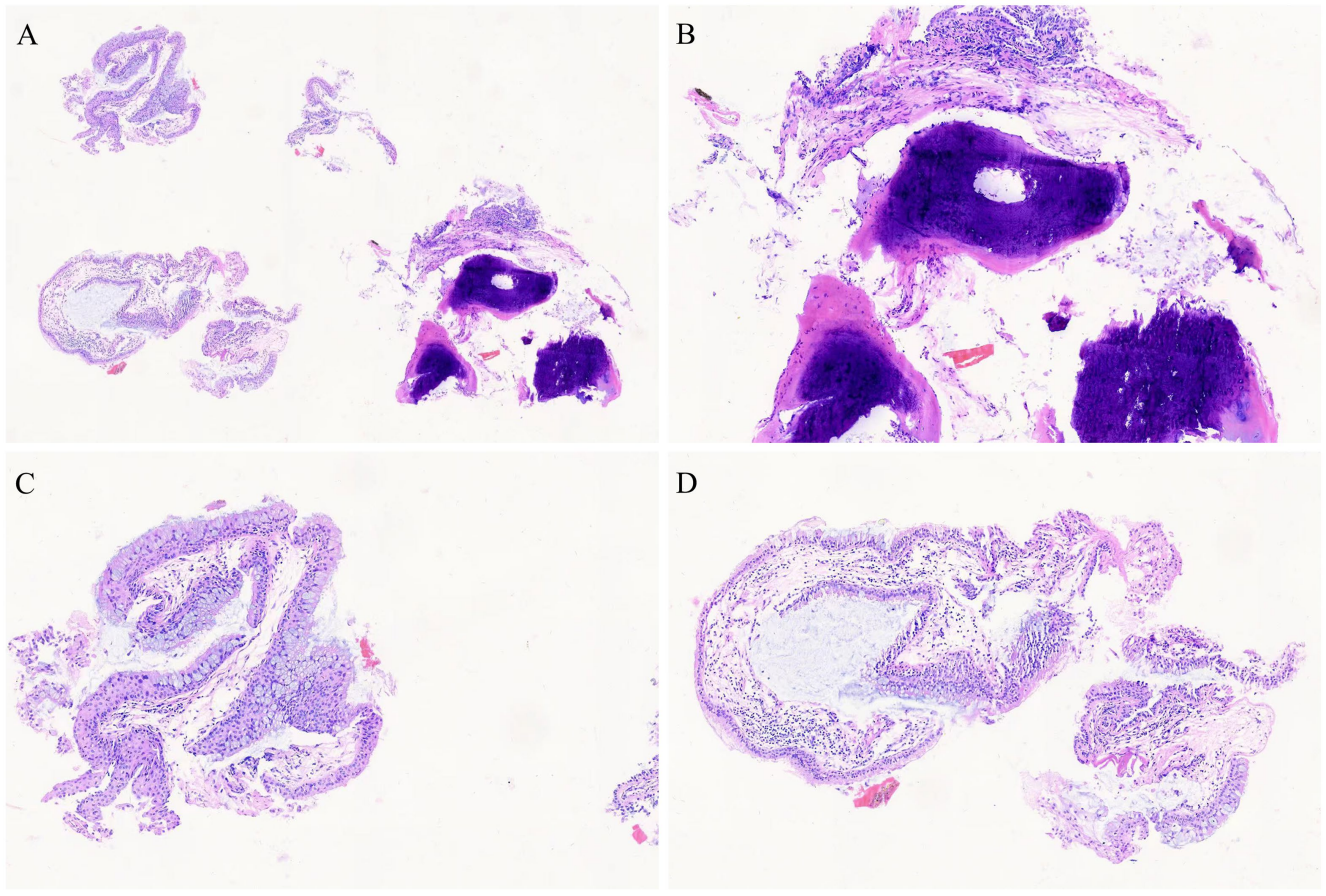
Pathological images (biopsy of these nodular tissues revealed ossification within the bronchial mucosa and focal surface epithelial squamous metaplasia, HE staining, **(A)** 4 × 10, **(B–D)** 10 × 10).

**Figure 3:**
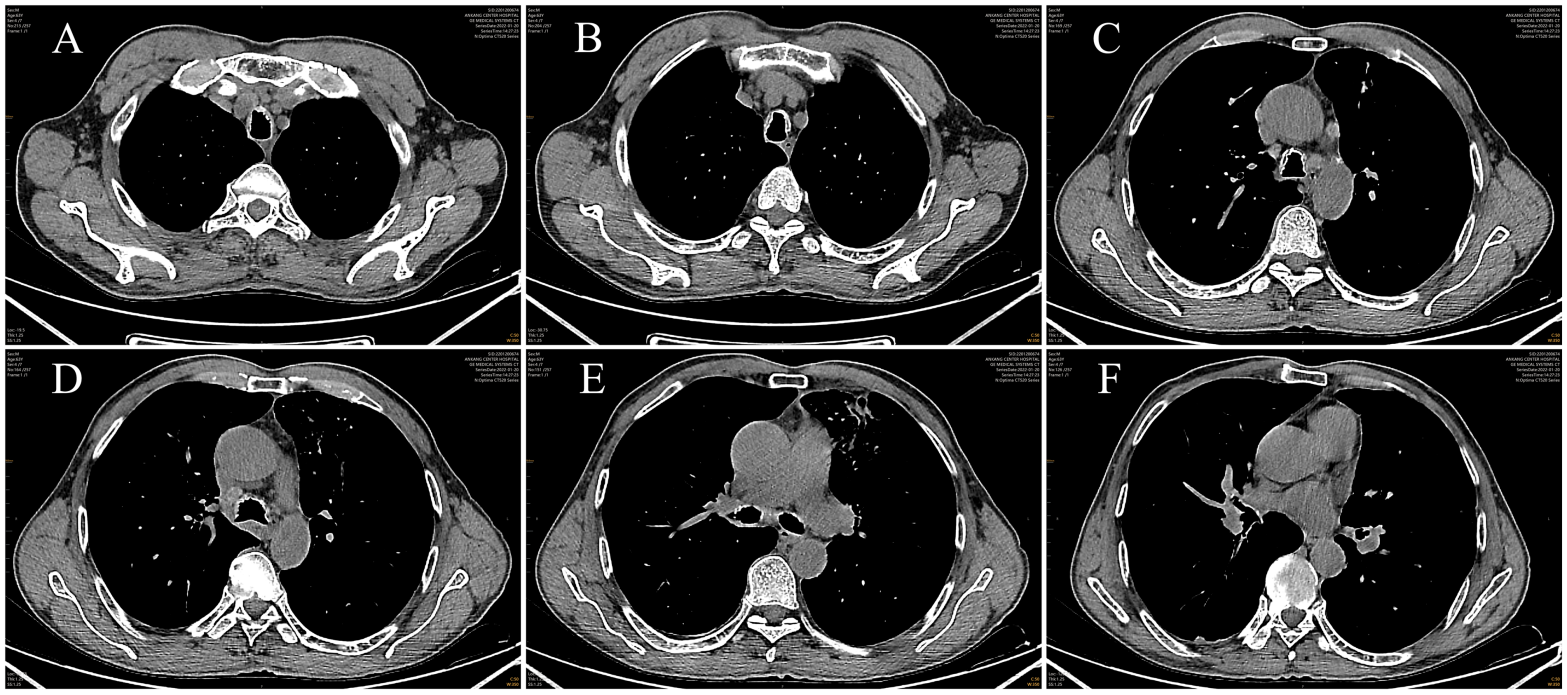
Images of mediastinal window from chest CT scan(a chest CT scan showed multiple calcified and non-calcified nodules protruding into the lumen of the trachea and main bronchi, with irregular luminal contours, sparing the posterior tracheal wall (membranous portion). (**(A, B)** Level Above the Aortic Arch, **(C, D)** Level at the Lower End of the Trachea, **(E)** Level of the Tracheal Bifurcation, **(F)** Level of the Right Middle Lobe Bronchus)).

Post-admission investigations included an electrocardiogram, pulmonary function tests, echocardiography, routine biochemical tests, lung tumor markers, and TBNK lymphocyte subset analysis, all of which were unremarkable. GeneXpert testing on bronchoalveolar lavage fluid was negative for tuberculosis. Cytological examination of the bronchoalveolar lavage fluid from the right upper lobe did not reveal any evidence of malignancy. A chest CT scan showed multiple calcified and non-calcified nodules protruding into the lumen of the trachea and main bronchi, with irregular luminal contours, sparing the posterior tracheal wall (membranous portion). Bronchoscopy revealed nodular lesions of unknown etiology in the trachea and bilateral bronchi (Figure 1). Biopsy of these nodular tissues revealed ossification within the bronchial mucosa and focal surface epithelial squamous metaplasia (Figure 2). Re-examination of chest CT scan: compared with the previous results, the lesions remain largely unchanged with no significant variation (Figure 3). The patient was subsequently treated with antibiotics (cefoxitin sodium 1 g q8h, levofloxacin hydrochloride injection 0.5 g qd), cough suppression and expectoration facilitation (Suhuang cough relief capsules 1.35 g tid, inhaled acetylcysteine solution 0.3 g tid).

The patient was discharged with a diagnosis of tracheobronchopathia osteoplastica and acute exacerbation of chronic bronchitis [differential diagnosis: (1) pulmonary mycosis, (2) HIV-associated pneumocystis pneumonia, (3) silicotic pulmonary fibrosis, (4) lung cancer]. Following the aforementioned treatment, the patient’s hemoptysis resolved, and his cough and expectoration improved. The patient requested discharge and was advised to enhance self-care, avoid exposure to cold, undergo periodic bronchoscopy, and follow up in the outpatient clinic. Two years later, telephone follow-up revealed that the patient had not experienced recurrent hemoptysis and only had occasional cough and expectoration.

## 3 Discussion

TO, a rare disease whose precise pathogenic mechanism remains enigmatic, is widely believed by scholars to be potentially influenced by multiple factors, including chronic infection, inflammation, congenital genetics, degenerative processes, chemical irritants, and mechanical injuries (4–10). Notably, persistent stimuli, such as chronic inflammation, continuously act upon the cartilaginous, bony structures, and fibrous connective tissue within the tracheal rings. This stimulation facilitates the gradual formation of chondromas and osteophytes, which subsequently develop into multiple nodules within the airway. Over time, these fibrous connective tissues undergo a process of chondrification and ossification, ultimately evolving into TO.

Furthermore, Virchow’s research has further elucidated the pivotal role of bone morphogenetic protein 2 (BMP-2) in the progression of this disease. In synergy with transforming growth factor beta 1 (TGF-β1), BMP-2 can induce chondrification of the submucosal connective tissue (11), thereby promoting the formation of chondromas from cartilage and osteophytes from bony structures. These lesions manifest as multiple hard nodules on the surface of the airway (12, 13). Under microscopic examination, these nodules exhibit a smooth, bony appearance (14). Due to the presence of these nodules, some of the luminal spaces become obstructed, impeding the drainage of secretions. Consequently, the structure and function of the bronchial mucosa become abnormal, ultimately giving rise to clinical symptoms such as cough, expectoration, and recurrent infections (15).

TO manifests clinically in a variety of ways, with many patients exhibiting no significant symptoms or only mild discomfort in the early stages of the disease. However, as the condition gradually progresses, some patients may experience persistent cough, while others may develop symptoms of dyspnea. In cases of infection, patients may also cough up purulent secretions. During the early asymptomatic phase, TO is highly susceptible to misdiagnosis or missed diagnosis. Nevertheless, thanks to continuous advancements in bronchoscopy and CT technology, clinicians’ understanding of TO has gradually deepened. The severity of this disease is closely related to multiple factors, including the presence of concomitant infection, the degree of airway stenosis, and the specific location of nodules within the airway. Therefore, once patients exhibit symptoms closely related to airway function, such as cough, wheezing, dry cough, hemoptysis, chest pain, and hoarseness, prompt diagnosis should be made through electronic bronchoscopy and CT three-dimensional reconstruction. It is important to note that if some patients do not receive active and effective treatment, the disease may progress to life-threatening conditions such as respiratory failure in its later stages.

The diagnosis of TO primarily relies on pathological biopsy, which serves as the gold standard for confirming this disease. In the case of this patient, the final diagnosis was also established through pathological biopsy. However, due to the hardness of the lesion site in TO, performing a biopsy requires a certain level of experience to ensure the acquisition of high-quality specimens. In terms of treatment, there are currently no specific therapeutic approaches targeting TO, and management primarily involves symptomatic relief based on the patient’s specific symptoms. Studies have suggested that inhaled corticosteroids may help slow the progression of the disease (16). With the continuous development of airway interventional techniques, interventional therapies including argon gas, plasma radiofrequency, microwave coagulation, electrocautery, and laser can be considered for patients with airway stenosis (17, 18). Additionally, stent placement is an effective method for addressing severe airway stenosis.

Overall, TO is a slowly progressive disease with a favorable prognosis. However, clinicians need to fully understand the characteristics of this disease, make an early diagnosis, and actively seek possible predisposing factors. Timely elimination of these predisposing factors is crucial for the treatment of patients.

## Data Availability

The original contributions presented in the study are included in the article/Supplementary material, further inquiries can be directed to the corresponding author.
